# Endodontic Treatment of a Maxillary Lateral Incisor with Two Roots; A Case Report with 6 Months Follow-Up

**Published:** 2014-12

**Authors:** Atefeh Hoseini, Abbas Abbaszadegan

**Affiliations:** a Dept. of Endodontics, School of Dentistry, Shiraz University of Medical Sciences, Shiraz, Iran.

**Keywords:** Maxillary lateral incisor, Tooth Root

## Abstract

Maxillary lateral incisors are widely known to be single rooted with one root canal. Although rare cases with root canal variations are being reported in many populations, the reports regarding Iranian population is extremely limited. In this report, we are presenting the endodontic treatment of a double rooted maxillary lateral incisor. These rare root-canal variations should be considered in pretreatment evaluations by clinicians who perform endodontic treatments.

## Introduction

Failure to eliminate the biological irritants from infected root canals with a careful preparation and obturation of canal space will eventually lead to an unsuccessful treatment. Furthermore, to access to success in endodontic treatments, a clinician must be aware of all root canal morphologies and their anatomical variations.[1]

Although the latest editions of endodontic textbooks assert that the maxillary lateral incisors have a single root in 100% of the cases, [2-3] reports of two-canalled [1, 4-8] or dual-rooted [1, 9-17] cases are also present in literature. Therefore, an experienced dentist should always consider the possibility of any existing variations such as extra canals or extra roots. Radiological evaluation is one of the most important diagnostic tools to help us in this issue.

In this report, the endodontic treatment and follow-up examination of an uncommon case of maxillary lateral incisor with two roots is presented. 

## Case Report

**History**

A 16-year-old female patient was referred to the endodontic department at Shiraz dental school with a chief complaint of slight pain in her maxillary right lateral incisor when biting. The patient also reported a history of swelling in her upper right anterior buccal mucosa during the past month.The patient had no abnormal medical history, complicating illnesses or history of trauma.

**Clinical Examination**

**Figure 1:**
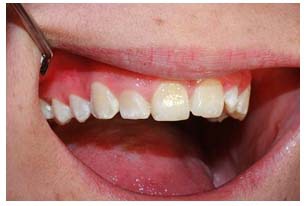
Intraoral view of the maxillary right lateral incisor

The extra oral examination revealed no abnormal findings (Figure 1). The intraoral examination showed no pathologic signs in the related soft tissue. The buccal vestibule of relevant tooth was also painless to palpation. The tooth had a mobility of 2+ and was painful to percussion.

Periodontal examination showed the presence of a periodontal pocket with the probing depth of 4mm. The crown of the affected tooth was intact; however it displayed a lingual pit above the cingulum. The result of the thermal tests was negative which confirmed a nonvital pulp. 

**Radiographic Examination**

**Figure 2:**
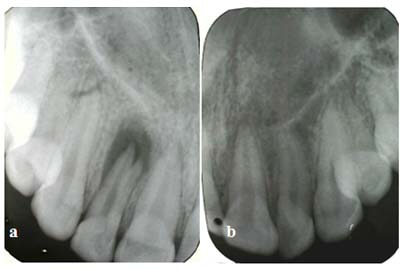
The maxillary right lateral incisor has two roots while the contralateral incisor is single-rooted a: Periapical radiographic image of the maxillary right lateral incisor b: Periapical radiographic image of the maxillary left lateral incisor

On the radiographic examination, two roots were detected in her maxillary right lateral incisor with an oval-shaped radiolucency around the roots (Figure 2a). It is also notable that the contralateral incisor was normal and had a single root (Figure 2b). With the aid of Clark's rule (same lingual, opposite buccal) the presence of a buccal and a palatal (the second) root was verified.

**Diagnosis**

The final diagnosis was made based on both clinical and radiographic examinations. The involved tooth had a primary endodontic lesion with necrotic pulp and acute apical periodontitis.

**Treatment options**

As a first treatment option, root canal therapy with several follow-up examinations was recommended to the patient. Consequently, in case of treatment failure, apicectomy will be performed. Tooth extraction and implant placement was another possible treatment option. The patient preferred to undergo root canal therapy.

**Treatment**

The tooth was isolated with a rubber dam. An access cavity was made from the lingual pit using high speed turbine and diamond fissure bur (Dentsply; Maillefer, Baillaigues, Switzerland) under cooling water spray. Since the teeth had a large orifice, the access cavity was prepared with an extra extension toward the palatal surface. This was done to make a straight access to the narrower root canal which was located palatally.

**Figure 3:**
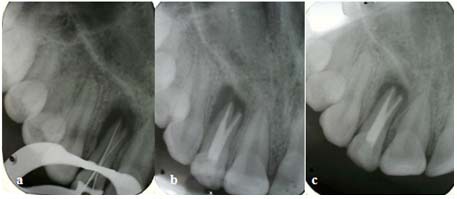
a: Working length determination X-ray b: Post obturation radiograph c: Six months follow-up radiograph

The absence of any vital pulp tissue or bleeding re-confirmed our initial diagnosis. Working length was then determined radiographically with a k-file # 35 (Mani, Japan) for the buccal canal and a k-file # 15 for the palatal root canal (Figure 3a). The working length measurements were also verified by Root ZX electronic apex locator (J Morita Corp; Kyoto, Japan). The root canals were prepared with passive step back technique up to the file #50 for the buccal canal and # 35 for the palatal root canal in the apical, using hand instrumentation. Gates Glidden drills (Dentsply; Maillefer, Baillaigues, Switzerland) were also employed to enlarge middle and coronal parts of the roots.

2.5% sodium hypochlorite was used as an irrigant during preparation. The root canals were dried with sterile paper points (Dentsply, Maillefer, Baillaigues, Switzerland). Calcium hydroxide powder (Henry Schein Company, Melville, NY) was mixed with sterile saline (Darupakhsh Co; Tehran, Iran) in a 6:4 powder/saline ratio to obtain a paste-like consistency. Then, the root canal was filled with Ca (OH)_2 _to the working length and tooth was coronally sealed with the temporary filling material (Cavit; 3M ESPE AG, Seefeld, Germany). A week later the patient was recalled. During reexamination, the tooth was completely in a normal condition with no tenderness to percussion.

The root canals were irrigated again, dried and obturated by lateral condensation technique using gutta-percha (Aryadent; Tehran, Iran) and AH26 sealer (Dentsply; Maillefer, Baillaigues, Switzerland).

A final radiograph was taken after completing the treatment (Figure 3b). Patient was again recalled after 1 week for permanent restoration with composite resin (EsteliteEQuick; Tokyama Dental Corp, Japan).

The 6-month follow-up examination of the patient revealed no clinical abnormalities. The tooth was completely asymptomatic and did not show any sign of mobility. The recall radiograph showed moderate bone formation in the periapical region (Figure 3c).

## Discussion

Disturbances in the normal development of Hertwig's epithelial root sheath can lead to some dental anomalies like grooves formation, dens in dente, gemination and fusion. These conditions may exhibit an appearance confused with double rooted teeth.[18]

In the present case, the pre-treatment clinical and radiological evaluations did not indicate the presence of any grooves or invaginations in enamel or dentin. Furthermore, the clinical examination apparently ruled out the conditions such as gemination or fusion. It is notable that gemination is where the double crown with a single root is present and the fusion is where a bifid crown with two root canals in a single root is evident.[16] Therefore, the current case is a rare case of double rooted maxillary lateral incisor without any particular morphological anomalies in the crown.

Although the main etiology of this variation is unknown, a disturbance in the development of Hertwig’s epithelial root sheath and consequent formation of a horizontal flap is construed. This hypothesis is a main reason for creation of an extra root.[19]Although, the etiologic factor of the pulp necrosis was also unclear, it might be attributed to the previous trauma that patient does not remember.

Considering the 6-month follow-up period, moderate bone formation was seen in the periapical region of the involved tooth. Moreover, the tooth had no abnormal clinical findings at the follow-up examination.It is notable that the surveys evaluating the outcome should be based on both clinical and radiological appraisal.[20] Besides, the evidences of complete healing status after initial treatment canbe display after one year follow-up period[21] and this rate will be increased over the time.[22] Therefore, the follow-up of at least one year is recommended to see the constant healing status.

To properly diagnose and handle the treatment of such cases, thorough radiological evaluations with Clark’s rule and taking parallel angle radiographs before and during the endodontic treatments are crucial and strongly recommended. Furthermore, using the new radiographic techniques such as cone beam computed tomography (CBCT) can also be very helpful in identification of anatomical features and variations of the root canal system.[17, 23] It is notable that CBCT is a high-resolution three-dimensional X-ray imaging approach and can be very useful in determining the precise location of extra roots.[23]

## Conclusion

Variation in the root canal is a possible finding during dental practice. A skillful clinician should have a comprehensive knowledge about any possible complexity or variation in root canal systems to be able to perform a sound treatment and achieve more predictable endodontic results. 
